# Program Evaluation of the *radKIDS®* Youth Personal Empowerment Safety Education Program

**DOI:** 10.1007/s40653-024-00618-5

**Published:** 2024-03-15

**Authors:** Deborah Johnson-Shelton, Stephen M. Daley, Jeff Gau, Naomi Canavan, Victoria E. Kress

**Affiliations:** 1 Oregon Research Institute, Springfield, Oregon, USA; 2 Saavsus, Inc, Eugene, Oregon USA; 3 radKIDS®, 9410 Harvest Acres Ct, Raleigh, NC 27617 USA; 4 University of Oregon, Eugene, Oregon, 97403 USA; 5 Youngstown State University, Youngstown, OH 44555 USA

**Keywords:** Bullying prevention, Child victimization, Social emotional learning, Trauma prevention, Evidence-based interventions, Mixed method surveys, Program evaluation

## Abstract

Developing sound evidence of program effectiveness can be difficult for many programs initiated by schools and communities, and impedes many beneficial programs from broader dissemination. This paper shares results of an evaluation approach used with a bullying and victimization prevention program with elementary school children called the ***radKIDS®* Personal Empowerment and Safety Education Program.** The purpose of this study was to examine indicators of initial effectiveness of the *radKIDS®* program for elementary school child safety skill development and instructor training to reduce child victimization and associated trauma and empower healthy psychosocial child development. The study involved 330 active radKIDS® instructors surveyed during two separate two-week periods, resulting in 148 completed questionnaires (45%). Instructors rated their perceptions of what children effectively learned in *radKIDS®*, the effectiveness of instructor training, and on Social Emotional Learning (SEL) competencies addressed in the program. Evaluation findings confirmed the theoretical model of the program, and that the developmental safety domains impacting children in *radKIDS®* differs from those in other bullying prevention interventions focused on SEL and other competencies. Recommended areas of improvement for the program included making training less time consuming and more flexible in delivery, provide more practice opportunities and time on skill acquisition during training, and increase supervision and guidance during program implementation.

**Supplementary Information:**

The online version contains supplementary material available at 10.1007/s40653-024-00618-5.

Programs designed and implemented by communities and schools face unique challenges in accruing evidence of positive program impacts and effectiveness. Unlike the systematic development and evaluations characterizing research-based interventions, most programs developed by community and school stakeholders are designed iteratively and at low cost to respond to local health problems or societal needs that may not clearly align with the focus, philosophy, or resource and support requirements of existing evidence-based programs (Aarons et al., 2011; Backer, 2000; Glissen & Green, 2006). In community contexts, programs often emerge and develop with community acceptance and use despite the absence of credible information on program effectiveness. The evidence base of many community-developed programs remains insufficient to justify investments in more rigorous high quality research designs. More experimental and implementation research is needed for extensive scaling up of community-developed programs..

In this paper the authors share an evaluation approach used to bridge this developmental evidence gap with an established program aimed at preventing child victimization and injury. The program evaluation consisted of a low-cost, mixed quantitative and qualitative online survey conducted with trained and experienced program instructors as a means of gathering diverse informant perspectives on the validity of the program’s instructional content and theory of change. The survey also asked instructors to identify shared perceptions of the program provided by other critical stakeholders including the student participants, school administrators and teachers, parents, and community members, and provide formative suggestions for curriculum and training improvements. Results of the evaluation were used to confirm the theory of change developed for the program, differentiate the targeted developmental domains impacting children in *radKIDS®* compared to other available bullying prevention interventions, and identify areas for improvement via multiple stakeholders’ perspectives to guide next steps in the program’s development and site-specific (e.g., school or community) implementation.

This study’s survey evaluation had three objectives: 1) confirm the theory of change developed for the program; 2) differentiate the targeted developmental domains impacting children in *radKIDS®* compared to other available bullying prevention interventions; and 3) identify areas for improvement via multiple stakeholder perspectives to guide next steps in the program’s development and site-specific (e.g., school or community) implementation.

The evaluation included eight research questions: 1) How were program components perceived in their effectiveness for child development; 2) How were program components perceived in their effectiveness for child development compared the those of Social Emotional Learning; 3) How were program curriuculum, training, and materials perceived by experienced instructors; 4) what aspects of *radKIDS*® did instructors perceive as having the most positively impact on child development; 5) What aspects of *radKIDS*® had been reported to instructors by key stakeholders (parents, school administrators, teachers, community members) as being most important; 6) What aspects of *radKIDS*® have stakeholders reported needing change; 7) What changes would instructors recommend for the program; and 8) what recommendations would instructors have for program dissemination.

## Background and Significance

Bullying, child victimization, and violence jeopardize the safety, health, and development of millions of American children each day (Geffner et al., 2021). About 30% of youth in the United States report moderate to frequent involvement in bullying in some capacity (Nansel et al., 2001). Children exposed to violence, abuse, or victimization can develop significant developmental trauma leading to mental health disorders such as anxiety, depression, and aggression (Hanish & Guerra, 2002; Hicks, 2021; Woods-Jaeger et al., 2019), lower self-esteem, and increased rates of suicide (Sourander et al., 2000). Youth are exposed to violence at such a high frequency that it can be seen as a “typical phenomenon” (Stokes & Jackson, 2016). Multiple studies have found that child victimization by peers, separate from family maltreatment or other adverse abuse, links to the development of psychosis such as hallucinations and delusions by late childhood and adolescence (Campbell & Morrison, 2007; Lataster et al., 2006; Trotta et al., 2013).

The developmental impacts of child victimization are extensive. Bullying is one example of an Adverse Childhood Event (ACE) which results in toxic stress (MacLochlainn et al., 2022). This toxic stress can harm child brain development as evidenced by problems with attention, complex planning, impulse control, decision making, and working memory (MacLochlainn et al., 2022). Bullied and victimized children have significantly higher school absenteeism, underachieve academically compared to their peers, engage less in prosocial activities, and are more at risk of school drop-out (Basch, 2011; Kochender & Ladd, 1996). Exposure to violence and victimization is also associated with the onset of substance abuse and delinquency (Anda et al., 2006) and violent behaviors during adolescence (Landsford et al., 2007; Stokes et al., 2016). Severe and frequent victimization of children contributes to cumulative risk and more adverse developmental outcomes both during childhood and adulthood (Gilbert et al., 2009; National Center for Missing & Exploited Children [NCMEC], 1999). Additionally, bullying and victimization extend to whole school populations and have recently been recognized as a continuum of behavior in which all school youth play a role (Espelage, 2016).

Trauma experienced during childhood can have long-lasting adverse effects on the child’s overall development and greatly impact how they relate to their environment and those around them. Bullying and abuse, both verbal and physical, can be experienced as traumatic for children, particularly for those children who have experienced past trauma, thus putting them at risk for developing emotional disorders and impaired psychosocial functioning (Vanderbilt & Augustyn, 2010). Substance abuse, anxiety, depression, aggression, and disordered eating behaviors are just a few of the struggles correlated with childhood trauma (Kress & Paylo, 2019). Examples of emotional abuse in the form of bullying include name-calling, verbal threats of physical harm, or fear due to feeling powerless against the perpetrator, most notable when these actions are repeated over time (Carney, 2008). Related to this idea, some believe that bullying perpetrators are maladaptively coping with their own trauma (e.g., witnessing physical violence, physical or sexual abuse) by modeling their own experiences and displacing them onto less threatening targets (Kelleher et al., 2008).

Children who have been victims of bullying or abuse can experience posttraumatic stress disorder (PTSD; Isdoe et al., 2021). Like all treatment of mental disorders and psychological dysfunction, with proper and early intervention, negative effects can be offset or prevented (Gaffney et al., 2021). Such interventions include trauma-focused cognitive behavioral therapy (TF-CBT), which have shown to enhance the psychosocial functioning of childhood trauma victims (Deblinger et al., 2011). Within schools, there are efforts to promote universal, trauma-informed care approaches for both preventing and intervening in the trauma exposure of children (Avery et al., 2021). Comprehensive, child victimization prevention programs may be able to contribute to these efforts and reach large populations of children to help ensure that children are not negatively impacted by victimization and do not experience future trauma that mirrors past trauma and abuse, further compromising their well-being and development. Even though tools and strategies have been developed to create safe, trauma-informed environments in schools, none have yet been implemented systematically or evaluated for benefits to schools or students (DeCandia et al., 2014).

Bullying studies were initiated over 50 years ago (Heinemann, 1972; Olweus, 1978, 1993, 2005; Olweus & Limber, 2010). While research on bullying prevention has grown significantly over the last two decades, overall incidences of physical, verbal, and relational aggression, and increasingly cyberbullying, continue to seriously challenge the safety of students and undermine the positive learning environments of schools (Boulton & Boulton, 2012; Finkelhor et al., 2014; Yeager et al., 2015). Approximately 25% of school-based prevention programs have been found to be somewhat effective in reducing peer victimization (McCallion & Feder, 2013). Meta-analyses conducted by Ttofi and Farrington (2011), Jiménez-Barbero et al. (2016), and Gaffney et al., (2018) on antibullying school programs have found modest to moderate reductions in bullying/victimization and changes in attitudes towards school violence. Gaffney et al. (2018) found that antibullying programs effectively reduced school-bullying perpetration by approximately 19–20% and school-bullying victimization by approximately 15–16%. Programs in this latter meta-analysis that had the largest effect sizes used age cohort designs. Overall, these reviews and meta-analyses found that the most effective programs were multicomponent, school-wide programs that sought to reduce bullying, victimization, and aggression across a variety of school settings (Abreu & Kenny, 2018).

Child bullying and victimization prevention programs primarily have used classroom level instruction and discussion formats, behavior monitoring and behavioral modification strategies with classrooms and/or individualized support for higher risk children, reinforcement of expectations for social and behavioral interactions by students, and school-wide deployment of policies and behavioral frameworks aimed at bullying prevention and positive social development of children (Diaz et al., 2021).

One curious feature of all of these programs aimed at bullying prevention (or the reduction of related behaviors) is the inattention given to the concept of individual child safety, an important aspect of any bullying prevention program. Thus, most successful, well-known school-based antibullying programs are not responding to nationally-recommended safety guidelines deemed essential for effectively protecting children from victimization and violence. These guidelines include a) providing concepts that help children identify bullying and victimization behaviors while introducing the skills they need to defend themselves in all types of situations, b) multiple program components repeated over years, c) involvement of parents and caregivers, d) reporting of incidences to adults, and e) the use of qualified presenters who use active participation, role play, behavioral rehearsal, and feedback processes in responding to adverse behaviors and/or situations (stopbullying.gov; NCMEC, 1999; Wurtele, 2009). Effective safety programs also are characterized by adequate intensity (e.g., providing an experience powerful enough to socially, emotionally, and cognitively engage children in new learning), duration, and scalable approaches for supporting age-appropriate safety knowledge and harm-resistant skills that children can generalize across multiple settings in schools (Finkelhor et al., 2014; Musu-Gillette et al., 2017).

The demand for effective bullying and violence prevention programs has grown over the last two decades with many states mandating school-based programs to help reverse violent and discriminatory behaviors among children in schools (Finkelhor et al., 2014; Musu-Gillette et al., 2017). From 1999–2010, 120 bills and amendments have been introduced by states and 49 states have passed antibullying legislation. The majority of these laws direct school districts to adopt antibullying policies (McCallion & Feder, 2013). Effective prevention programs are being sought to fill these needs.

The *radKIDS®* **Personal Empowerment and Safety Education Program** (https://www.radKIDS.org/) is a universal, community-based program for protecting children from violence and victimization, which was developed in response to national recommendations for effective safety education. *radKIDS®* is aimed at empowering children to protect themselves against victimization by both peers and adults, including verbal, physical, relational, and cyber abuse. The program is unique in its use of behavioral skill training to help children establish personal boundaries for safety, focus on critical thinking skills to respond to threats of danger, and develop age-appropriate coping strategies for dealing with current and past victimization. The program emphasizes strengthening child self-assertiveness skills for defending themselves and others (when bystanders), learning communication skills for reporting incidences to parents and adults, and developing positive peer relations and respectful interactions during conflict, with an overall emphasis on growing child self-worth—the program’s cornerstone for personal safety and healthy development. *radKIDS®* behavioral safety skill training provides students with activity-based risk reduction skills and safety planning that generalize across all school settings (e.g., classroom, playground, hallways, bathrooms, playgrounds, gyms, and lunchrooms). The program also provides important skill training on abduction avoidance, establishing personal space and personal touch safety to prevent sexual abuse and assault, injury prevention and home safety planning (including behavioral training found to be effective for gun safety; Miltenberger et al., 2004), and skill training for safe transport between home and school.

For over 21 years, *radKIDS®* has been conducting behaviorally based bullying and violence prevention in schools and communities and has trained over 4,000 instructors and taught over 300,000 children in preschools and elementary schools. In *radKIDS®*, elementary school children develop individual safety plans applicable to a comprehensive menu of daily living environments and situations. These personalized safety plans and the simulation techniques used in the program engage children directly in the processes of preventing, resisting, and avoiding bullying, violence, and abuse. The program involves a minimum of 10 hours of interactive group classroom educational sessions combining instruction, discussion, and active behavioral skill training drills. The curriculum includes six domains of child safety training: (1) an introduction to the program’s philosophy, the three *radKIDS®* rules, and core safety planning components; (2) safety and skill development to avoid bullying at school; (3) safety plans and skills to prevent victimization and injury at home; (4) out and about safety in neighborhoods and the community; (5) awareness and skills for preventing and responding to sexual assault; and (6) learning how to avoid and resist abduction. A final celebration is held at the end of the program where students receive certificates of completion. Parents are encouraged to attend *radKIDS®* sessions with their child, support their child in completing homework and developing individual safety plans, and practice and grow in safety skills. Caregivers also receive a family manual (available in Spanish and English) that shares all the content of the program and are invited to participate in complete program training to become *radKIDS®* certified instructors.

*radKIDS®* instructors, who lead children in the group-based learning activities, undertake a 40-hour in-person training certification and licensing program. Instructors are typically classroom teachers, school counselors, physical education teachers, health teachers, law enforcement officers assigned to work with schools, community-based professionals, and concerned and interested school parents.

## Theory of Change

In preparation for this evaluation, the study’s researchers and program Chief Executive Officer (CEO) held multiple discussions and reviewed literature to identify the underlying theories guiding and supporting the program’s framework for child multisector (school, home, community) safety skill development, and specify a theory of change model for *radKIDS®* that would be examined in the evaluation.

The guiding theories of the *radKIDS®* program are derived from the tenets of ecological systems theory, social cognitive theory, situated learning theory, and behavioral skill training. In ecological systems theory (EST), individual development and change cannot adequately be understood without recognizing the context, or ecological niche, in which the individual is embedded (Broffenbrenner, 1986; Broffenbrenner & Morris, 1988). In the case of children, their ecological niche includes their families, schools, and the larger niches that surround their families and schools (i.e., communities and broader society (Davison & Birch, 2001). The adaptations children need to make within their unique ecological niches makes the development of flexible safety planning and skill application essential for personal safety.

Social Cognitive Theory (SCT) is the notion of “reciprocal determinism,” the suggestion that the environment, the person, and behavior are continually interacting. That is, individuals’ behaviors, such as a choice to engage (or not engage) in aggressive or risky behavior, are influenced both by the environment in which they live and their personal characteristics. This influence is ongoing and dynamic, with all three elements (the characteristics of the individual, the individual’s behavior, and the individual’s environment) mutually interacting. According to SCT, individuals are active agents who interpret and interact with their environment. Changes in knowledge, attitudes, and behaviors may occur through observational learning, through direct or vicarious reinforcement, either extrinsic or intrinsic, and through changing expectations. radKIDS focuses on children gaining the locus of control over their own safety and learning by recognizing and developing trusted relationships and communication patterns with peers and adults. Children learn to recognize safe and unsafe behaviors and places for personal interactions through instruction, observation, and applied practice-based skill learning.

Situated Learning Theory (SLT) is the view that learning as it occurs naturally (as compared to in the classroom) is embedded or “situated” in activity, context, and culture. Therefore, knowledge needs to be presented in authentic contexts that reflect the situation applicable to the learning—unlike most classroom learning, which can be typically more abstract and removed from context. According to Lave and Wenger (1991), in SLT, social interaction and collaboration form essential components of situated learning. In this way, learners become involved in a “community of practice” that embodies certain beliefs and behaviors to be acquired. Lave and Wenger argue that individuals begin at the periphery of a community and move to its center as they become more active and engaged within the community and develop expertise in the surrounding culture.

Behavioral Skill Training (BST), derived from the field of Applied Behavior Analysis, is a teaching and learning strategy that consists of a combination of *behavioral* techniques for both teachers and learners. BST involves four processes involving instruction, modeling, rehearsal, and feedback for the acquisition of new skills described here (Miltenberger, 2004):

- *Instruction*. Provide a description of the skill, its importance or rationale, and when to and when not to use the skill. Repeat this step as necessary.
- *Modeling*. Show how to perform the skill.
- *Rehearsal***.** Allow participants multiple opportunities to practice the skill. The trainer records/identifies correct and incorrect responses.
- *Feedback***.** Positive praise for individuals responding correctly and some form of corrective feedback for incorrect responses.

Modeling of behavioral skills is more effective if demonstrated with high levels of integrity (Miltenberger, 2004) and if multiple examples are provided of the targeted skills (Moore & Fisher, 2007). Additionally, research has found that rehearsal is ineffective without feedback (Ward-Horner & Sturmey, 2012). radKIDS® uses Behavioral Skill Training heavily in simulating risk situations in drills for skill building which aim at transmitting effective child safety and help-seeking behaviors.

All four of these theoretical tenants are foundational to the radKIDS® program which are made operational with the three radKIDS® principles which guide all instruction and skill building in the program:

1. No one has the right to hurt me, because I am special;
2. I don’t have the right to hurt anyone else, including myself, unless someone is trying to hurt me and then I have the right to stop them; and
3. If someone hurts me, it is not my fault. I can tell and keep telling until someone helps me.

**Fig. 1:**
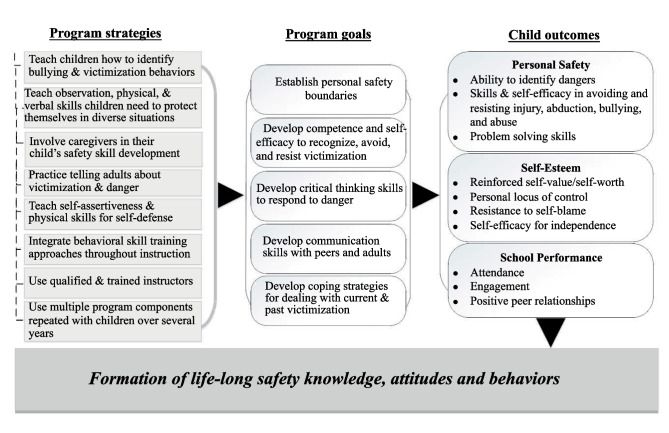
radKIDS® change mechanisms in transferring knowledge, attitudes and skills in child safety development

While the four principles of EST, SCT, SLT, and BST were determined to guide the approach and content of *radKIDS®,* the evaluation/program team developed a conceptual model for the theory of change in *radKIDS®* that links specific learning strategies of the program to the developmental goals and anticipated outcomes for children, as shown in Fig. 1. The eight program strategies shown in column one represent the national guidelines for child safety education and training that are incorporated into *radKIDS®* teaching and learning content and skill training processes*.* The five program goals depicted in column two represent the core child competencies that the program’s active learning strategies are aimed at developing in school-aged children. The three areas of child outcomes depicted in column three focus on the program’s priority in developing children’s personal safety, followed by related dimensions of growth in child self-esteem and enhanced school performance.

## Method

### Survey Design

A research team at Oregon Research Institute designed and assessed a 58-item a non-standardized online survey instrument with 148 instructors located in 26 states during the summer and early fall of 2018. The purpose of the survey was to 1) clarify the educational and developmental theory of the established child safety education and instructor training approaches, 2) solicit feedback on effective and ineffective program components, and 3) gather feedback that instructors had received from key program stakeholders (school staff, parents, community members, and children trained in the program) on program impacts, program satisfaction and desired improvements. By drawing on the views and experiences of *radKIDS®* instructors, this evaluation sought to leverage instructor’s implementation expertise to develop a clearer systems framework and theoretical model for the program and guide next steps in program development and implementation activity**.**

The 58-item survey included eight demographic questions on the professional background of instructors, the state in which they taught *radKIDS®*, how long they had taught the program, the numbers of groups and children they had instructed, the ages of children taught, and instructor educational background and sex. The survey also included items designed to ascertain what children effectively learned in the *radKIDS®* program (e.g., identifying bullying behavior) with a 5-point response option (1 = *strongly disagree*, 5 = *strongly agree) *for 23 items focused on *radKIDS®* specific knowledge and skills and 12 items related to social and emotional learning (SEL) competencies adapted from the Collaborative for Academic, Social, and Emotional Learning (www.casel.org). The intention was to examine how *radKIDS®* instructors would rate the impacts of the program on the target developmental goals compared to indicators of social and emotional learning competencies. Scree plots and eigenvalues from a principal components analysis supported a one component solution for the 23 specific and knowledge items (eigenvalue = 18.4, 74% of variance explained; Cronbach’s alpha = 0.98) and the 12 SEL items (eigenvalue = 7.9, 72% of variance explained; Cronbach’s alpha = 0.96). Additionally, the survey included 14 items pertaining to *radKIDS®* training and instructor support (e.g., training provided enough time for skill practice for you to feel able to begin teaching the program to students) with a 5-point response option (1 = *strongly disagree*, 5 = *strongly agree*). Other questions on the survey included items on instructor satisfaction with training and program materials (1 = *strongly disagree*, 5 = *strongly agree*), their estimate of the percentage of children they taught who had experienced some form of violence, bullying, or victimization prior to receiving the program, and the level of support instructors had experienced within the school setting for delivering the program. The survey also included five open ended questions on (1) what aspects of *radKIDS®* instructors had observed to have the most impact on children’s development, (2) how *radKIDS®* Instructor Training and Certification could better prepare and support instructors and be improved for delivering the program, (3) aspects of the program described by the aforementioned stakeholder groups as most important for child personal, social, and emotional development, (4) the recommendations stakeholders had described as most important for improving *radKIDS®*, and (5) how the program could be more broadly implemented in schools and communities.

### Data Collection

Online surveys were distributed by email through Qualtrics over two 2-week intervals to instructors identified by the CEO as either long-term instructors (engaged more than one year in the program) or trained and teaching at the time of the assessment. The two distributions occurred during the summer and fall of 2018. In all, 330 instructors received the survey and 148 responded to questionnaires over the two, 2-week periods (a 45% response rate).

### Data Analysis

Participant data and closed ended survey variables were summarized using descriptive statistics, and paired *t*-tests were used to examine potential differences in *radKIDS®* safety knowledge and skills targeted competencies for children compared to SEL competencies. The point-biserial correlation was computed as a measure of effects size and follows the convention 0.14 small, 0.36 medium, and 0.51 large (Rosenthal & Rosnow, 2008).

Qualitative responses to open ended survey questions were exported from Qualtrics into Excel for data analysis. The lead researcher in the study and a research assistant completed content analysis of written responses (Patton, 2015). Data was first cleaned of responses not relevant to questions posed, then responses were coded and organized into themes to interpret similarities and differences in perspectives and to develop more nuanced constructs for categorical codes. The two researchers independently coded question responses to develop thematic categories, and then reviewed and completed a final coding schema for each question. Responses for each question were then coded by each researcher, then reviewed for mutual agreement. Coded responses were then ordered into frequencies to determine the strength of theme patterns that emerged among responses for each question. The four open-ended questions analyzed and reported in this study include 1) What aspects of *radKIDS®* have you personally observed to have the most positive impact on children’s development? 2) What aspects of *radKIDS®*, if any, have been described as most important for child personal, social, emotional development? (By school administrators? By teachers? By students? By parents? By the community?), 3) What aspects of *radKIDS®* have program stakeholders described to you as most important for additions to the program, enhancements or areas of improvement? and 4) What aspects of *radKIDS®* do you personally believe need improvement? (Please describe why and, if possible, how you would suggest improvements being made).

### Findings

#### Participant characteristics

The majority of instructor participants in this study were female (80%), and represented diverse professional backgrounds (Table 1). The largest proportion of instructors were either parents (24%) or law enforcement personnel (23%). Respondents from other backgrounds (16%) included martial artists, social workers, community health workers, principals, community coordinators, afterschool program staff, and grandparents. Physical education teachers (11%) and school counselors (8%) were also highly represented.

**Table 1:** Instructor demographics

		N	%
*Sex*			
	Male	29	20%
	Female	119	80%
*Instructor background*			
	Parent	35	24%
	Law enforcement	34	23%
	Other	24	16%
	Physical education teacher	16	11%
	School counselor	12	8%
	Regular education teacher	7	5%
	Teaching assistant	7	5%
	Community professional	7	5%
	Early childhood education teacher	6	4%
*Instructor Education*			
	High school degree	8	5%
	Some college	20	14%
	2-year associate degree	15	10%
	4-year college degree	58	39%
	Master’s degree	42	28%
	Doctoral degree	5	3%
*Years teaching radKIDS®*			
	< 1 year	13	9%
	1—< 3 years	33	22%
	3—< 5 years	39	26%
	5—< 7 years	28	19%
	7—< 9 years	10	7%
	≥ 9 years	25	20%
*# children’s classes taught*			
	1—3 groups/classes	17	12%
	4—6 groups/classes	18	12%
	7—9 groups/classes	12	8%
	10—12 groups/classes	11	8%
	13—15 groups/classes	14	10%
	> 15 groups/classes	74	51%
*# children taught over time*			
	< 50	13	9%
	50—99	18	12%
	100—199	26	18%
	200—299	12	8%
	300—399	17	12%
	≥ 400	62	42%
*Ages of children taught*			
	Preschoolers	26	20%
	5 – 7 year olds	108	82%
	8 – 12 year olds	117	89%

Instructor longevity and experience in implementing the program were important in providing valid feedback on survey questions. Forty-six percent of the responding instructors reported teaching *radKIDS®* for five years or more. Only 9% of respondents reported teaching the program less than one year. Over half of the instructors had taught 15 or more classes and 54% indicated they had taught 300 or more children in the program. Respondents were broadly dispersed geographically across 26 states in the U.S. The largest proportion of respondents resided in Utah (30%), Texas (13%), Florida and Georgia (12% each), and Massachusetts (7%).

#### Instructor rating of effective program components on child development

A key goal in this evaluation was determining instructor’s perception of the effective components of the program. We asked instructors to rate 23 statements presenting child safety learning objectives in *radKIDS®* and 12 social emotional learning objectives using a 5-point scale from *strongly disagree* (1) to *strongly agree* (5). Table 2 presents the total percentage of responses by category and the mean score for each item. The highest rated items are related to the primary goals of *radKIDS®.* For example, instructors reported a mean score of 4.8 on children learning to “identify unwanted touching,” developing “effective skills to ask for help/protection when needed,” and learning to “make decisions based on safety concerns.” The program was also rated very highly in teaching children how to “recognize and resist abductions” and “be safe when out and about in the community.” Responses confirmed that instructors viewed the program as positively impacting students in intended areas of growth and development. The lowest scoring item in the child safety development variables was children learning to “resist or avoid bullying as a bystander.”

**Table 2:** Instructor rating of what children learned effectively in the program

*Domain* / Item	Strongly disagree *%*	Slightly disagree *%*	Neither agree nor disagree *%*	Slightly agree *%*	Strongly agree *%*	*Mean*
*radKIDS® specific knowledge and skills*						
Identify unwanted touching	1	–	2	6	90	4.8
Develop effective skills to ask for help	1	–	2	15	82	4.8
Recognize and resist abductions	1	–	2	15	82	4.8
Make decisions based on safety concerns	1	–	1	18	80	4.8
Resist or stop unwanted touching	1	–	2	17	79	4.7
Be safe when out and about in the community	1	1	1	20	77	4.7
Understand and resist trickery behavior	1	–	3	17	79	4.7
Identify bullying behavior	1	2	1	16	79	4.7
Recognize/use family, school, community resources for safety	1	1	3	21	75	4.7
Use *radKIDS®* rules in their daily lives	1	–	4	19	76	4.7
Be safe at home	1	–	4	21	74	4.7
Resist physical victimization from adults	1	–	5	20	74	4.7
Wear seat belt, bike helmet during transport	1	1	4	19	74	4.7
Avoid physical victimization by adults	1	–	6	18	74	4.6
Prevent home injuries for themselves	1	–	4	25	71	4.6
Resist physical victimization from peers	1	–	4	25	69	4.6
Resist bullying others	1	1	4	26	69	4.6
Recognize, avoid risky community situations	1	1	4	24	69	4.6
Avoid physical victimization from peers	1	–	4	27	67	4.6
Develop personal safety plans	1	–	4	28	66	4.6
Resist or stop bullying by others	1	–	4	30	65	4.6
Use safety behaviors at home	1	–	7	25	67	4.6
Resist or stop bullying as a bystander	2	2	11	34	51	4.3
*Social Emotional Learning Competencies*						
Accurately assess their own personal boundaries, safety feelings, interests, values, and abilities	1	1	3	30	65	4.6
Maintain well-rounded sense self-confidence	1	1	7	37	54	4.4
Maintain healthy relationships with others	1	3	8	37	51	4.4
Make decisions on standards of conduct	1	1	14	30	54	4.4
Make decisions based on respect for others	1	2	9	39	49	4.3
Contribute to school/community well-being	1	1	13	32	53	4.3
Make decisions based on likely consequences of actions	1	4	13	34	49	4.3
take perspective of and empathize w/others	4	4	10	38	45	4.2
Control impulses for aggression	1	6	14	44	36	4.1
Prevent, manage, resolve interpersonal conflict	1	6	16	40	36	4.0
Express emotions constructively	2	5	17	44	33	4.0
regulate emotions to handle stress	2	8	17	38	35	4.0

Instructor ratings of dimensions of social emotional learning effectiveness of the program scored lower as a group than the *radKIDS®* specific learning variables. The average social emotional learning score was 4.23 (*SD* = 0.76) and the average child safety training score was 4.65 (*SD* = 0.59). The difference in scores was statistically significant (*t*[143] = 9.41, *p* < 0.001) and the difference was associated with a large effect (*r* = 0.62).

#### Instructor rating of program training, materials, and supports

The program was viewed positively for curriculum content (Table 3). Instructors rated highest the clear development of learning components used for instruction in the program. The lowest rated program elements included the lack of involvement of school administrators and staff in reinforcing program learning.

**Table 3:** Instructor rating of training, program materials and supports

Questionnaire Items	Strongly disagree (*%*)	Slightly disagree (*%*)	Neither agree nor disagree (*%*)	Slightly agree (*%*)	Strongly agree (*%)*	*Mean*
Learning components for students were clearly developed for instructional purposes	0	0	4	21	75	4.7
Lesson syllabi provided in your certification training were easy to understand and use	0	2	4	27	68	4.6
You felt you had adequate instructional support from the organization to be successful in delivering the program	0	6	6	18	70	4.5
Videos to help instructors lead physical skill training and drills on the fly were helpful and of sufficient quality	0	2	7	28	63	4.5
The *radKIDS®* manual was organized clearly for you to follow and use for facilitating the program with students	0	5	6	24	65	4.5
Videos were easy for you to access and use	< 1	2	8	24	65	4.5
Materials for families were effective in engaging them in their child's safety learning and development	0	6	9	23	62	4.4
Homework exercises for students were well developed and useful to children in reinforcing lesson content	0	3	15	25	57	4.4
You were comfortable in managing logistics for implementing the program in the school	0	5	15	26	55	4.3
Training provided enough time for skill practice for you to feel able to begin teaching the program to students	4	8	2	35	51	4.2
Adequate resources were available to help respond to behavioral, or abuse needs of students if issues arose	0	5	18	29	48	4.2
You had adequate training and support to work with parents	2	8	13	28	50	4.2
You were able to cover all intended program content with students within suggested time frames from training	3	10	6	38	43	4.1
School administrators and staff knew how to properly reinforce *radKIDS®* rules and practices in the school	3	13	20	33	313	3.8

#### Aspects of *radKIDS*® instructors reported to most positively impact child development

A core goal in the survey was to ascertain the aspects of the program instructors had *“*personally observed to have the most positive impact on children’s development.” This was an open-ended question and responses were analyzed and coded into 12 constructs of child developmental impacts shown here.

1. Confidence: growth in student confidence in themselves generally and, specifically, in relationship to taking care of themselves and protecting themselves from harm;
2. Empowerment: growth in understanding how to take personal responsibility for themselves;
3. Self-agency for protection: increase in assuming locus of control for their own safety planning and behaviors instead of relying on adults;
4. *radKIDS®* rules: the three *radKIDS®* rules were considered a core aspect of the program in giving children a rubric for interpersonal safety: 1) no one has the right to hurt me because I am special, 2) I do not have the right to hurt anyone, including myself, except for protection, and 3) it’s not my fault, so I can keep getting help until someone listens;
5. Safety planning and skills: development of safety planning in response to their individual context and related observation, critical thinking, and physical skills training;
6. Victimization prevention: stopping abduction, sexual assault, adult abuse, and bullying in children’s lives;
7. Voice: learning to be loud verbally and physically, but also learning to assert themselves to be safe;
8. Right to say “No” to adults: understanding that they have full permission to reject adult overtures that are inappropriate for them as children and harmful or uncomfortable as a person;
9. Identifying danger: growth in ability to scan environments and people for danger;
10. Self-worth: growth in realizing they are each special and the most important person in their life and no one has the right to hurt them;
11. Child–adult connection and communication: introducing positive adult relationships into children’s lives and developing communication with trusted adults; and
12. Less aggression: reducing child fighting and aggression, as well as creating stronger order in the classroom.

**Fig. 2:**
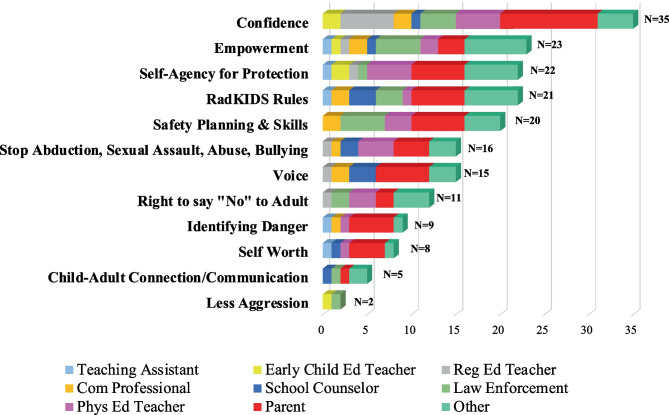
Aspects of radKIDS instructors identified most positively impacting children’s development

We calculated the frequency of responses for each of the 12 positive program impacts and ordered them by background categories of instructors (Fig. 2). Each construct identified by instructors aligned with the instructional goals of *radKIDS®* and confirmed the program’s theory of change model for children’s development. Growth in child confidence was reported the most frequently (*n* = 35) and was noted by all instructor types, except teaching assistants. The program’s empowerment of children, facilitation of child self-agency for personal safety, effectiveness of the three *radKIDS®* rules in helping children frame their own and others’ behaviors for safety, and the safety planning and skills children learned in the program were also strongly identified as features of the program most positively impacting child development.

The least frequently mentioned item, but still identified, was the reduction of aggressive behavior and creating more order in the classroom. (Instructor written comments on this question are shared in supplemental material, Supplementary file 1.)

#### Aspects of *radKIDS*® most important to other stakeholders

The evaluation sought to take advantage of the direct contact instructors have with key stakeholders in the program to systematically assess their perspective on the benefits of the program to children. The survey asked the open-ended question of instructors “*What aspects of radKIDS®, if any, have been described as most important for child personal, social, and emotional development?”* for school administrators, teachers, students, parents and the community (Table 4). More instructors responded with comments for parents (n = 90) and students (n = 89) than other groups. However, many instructors provided multiple values for each stakeholder group; for instance, instructors attributed 101 counted program features deemed most important by school administrators compared to 64 for the community. Also, some instructors taught in community settings and not in schools so they could not provide insights into school administrator or teacher perceptions of program benefits for students. These instructors, though, sometimes had more interaction with parents and also received feedback from both parents and community members about the need for *radKIDS®* to be taught in schools to benefit all children in the community (see instructor comments in Supplemental Materials, Supplementary file 1).

**Table 4:** Aspects of *radKIDS®* most important to stakeholders for children in the program

Coded Items	**School Administrators (*n* = 77)** **# %**	**School Teachers (*n* = 72)** **# %**	**Students (*n* = 89)** **# %**	**Parents (*n* = 90)** **# %**	**Community (*n* = 73)** **# %**
Bullying	23 (23%)	23 (33%)	5 (6%)	7 (8%)	4 (6%)
Child safety	17 (17%)	14 (20%)	16 (18%)	19 (20%)	12 (19%)
Behavior management	5 (5%)	8 (11%)	–	–	4 (6%)
Child self-confidence, self-worth, empowerment	9 (9%)	10 (14%)	36 (40%)	9 (10%)	4 (6%)
School safety, communication, culture	25 (25%)	11 (16%)	–	–	3 (5%)
Sexual abuse prevention	3 (4%)	3 (4%)	–	6 (7%)	5 (8%)
Abduction prevention	1 (1%)	1 (1%)		5 (6%)	4 (6%)
School PR & requirements	4 (4%)	–		–	–
Physical Skills/Drills on the fly	4 (4%)	–	13 (14%)	15 (17%)	–
Children learn their own safety skills/contexts	10 (10%)	–	13 (14%)	20 (22%)	6 (9%)
Children had fun	–	–	7 (8%)	2 (2%)	–
Family manual, relationships, communication	–	–	–	10 (11%)	–
Safer communities/crime prevention/less victimization	–	–	–	–	18 (28%)
Support & involvement with children	–	–	–	–	4 (6%)
TOTAL RESPONSES	101	70	90	93	64

School administrators were described as particularly concerned about school safety, clear communication on safety issues, safe school culture (25%), bullying prevention (23%), and child safety (17%). Responses indicated that teachers also were primarily interested in the program’s promotion of school safety, but, most importantly, around bullying prevention (33%). Concern for child safety was also high (20%). Unlike responses for other stakeholder groups, the most salient feature of *radKIDS®* for students was their enjoyment of the program—the content, the learning processes, and the personal outcomes. In terms of their personal, social, and emotional benefits, instructors felt students mostly valued the self-confidence, empowerment, and self-worth they gained from the program (40%). Learning how to be safe (18%), the physical skills and drills on the fly (14%), and acquiring the safety skills that applied to their own individual life (14%) were the attributes of the program that instructors felt resonated the strongest for students. Parents expressed considerable support for the program, particularly in its focus on child safety. More than any other group, however, instructors described parents as particularly interested in the program’s focus on equipping children to learn their own safety skills (22%) so they would not be reliant on parents or other adults to protect them from harm. According to instructors, the most important aspect of *radKIDS®* for community members was making the community safer, helping to prevent crime in the long term with this investment in children, and reducing victimization (28%). Improving child safety in the community was also very important (20%).

#### Aspects of *radKIDS*® needing change according to stakeholders

In a follow-up question to the one described above, we asked instructors “*What aspects of radKIDS® have program stakeholders described to you as most important for additions to the program, enhancements, or areas of improvement?”* For school administrators and teachers, the most frequently suggested improvement was to somehow teach the program more quickly or fit the program better into existing schedules (25% and 29% respectively; Table 5). Teachers had also mentioned more content on bullying prevention and more opportunities for them to be trained in the program, even with an abbreviated training so they would be better able to support the curriculum being taught by instructors. Students had consistently mentioned wanting more of the program generally, but they also wanted more time devoted to drills and the active simulations in the program. In fact, 65% of the changes recommended by students focused on more activity in the program. Parents expressed the desire to see the program taught more often and to train up more instructors to be able to expand program offerings (29%). This same desire was consistently mentioned by community members. Almost half of the improvement recommendations by the community were focused on more opportunities for program delivery with more trained instructors (46%). Multiple stakeholders had suggested updating the curriculum with more training on social media safety and adding curriculum on active shooters in the schools, which are currently being further developed.

**Table 5:** Stakeholder recommended improvements for *radKIDS®*

Coded Responses	School Admin *n* = 19 *# %*	School Teachers *n* = 20 *# %*	Students *n* = 35 *# %*	Parents *n* = 28 *# %*	Community *n* = 22 *# %*
Teach program more, train more instructors	2 (8%)	2 (8%)	5 (14%)	9 (29%)	12 (46%)
Provide more on bullying, types of bullying	2 (8%)	4 (17%)	1 (3%)	2 (6%)	–
More info about the program to school/community	1 (4%)	1 (4%)	–	1 (3%)	2 (8%)
Integrate *radKIDS®* in schools, part of curriculum	–	2 (8%)	–	2 (6%)	2 (8%)
Update curriculum on internet, phones, texting	3 (13%)	2 (8%)	1 (3%)	5 (16%)	1 (4%)
Less talking & more activity, drills, physical skills	2 (8%)	–	24 (65%)	2 (6%)	–
Lessen time for instructor training	2 (8%)	–	–	–	–
Add curriculum on active shooter	2 (8%)	2 (8%)	1 (3%)	1 (3%)	1 (4%)
Make the program last longer	1 (4%)	–	3 (8%)	2 (6%)	–
Teach program more quickly, fit to scheduling	6 (25%)	7 (29%)	–	–	–
Provide shorter training (< 40 h)	1 (4%)	4 (17%)	–	–	–
Find ways to support program costs for schools/communities	1 (4%)	–	–	–	3 (12%)
Provide more time for kids to talk out situations	–	–	2 (5%)	–	–
Make family manual simpler, engaging parents	1 (4%)	–	–	4 (13%)	–
Make *radKIDS®* available/required for all students	–	–	–	3 (10%)	5 (19%)
TOTALS	24	24	37	31	26

#### Aspects of *radKIDS*® recommended for change by instructors

This evaluation also posed the formative question to instructors: *“What aspects of radKIDS® do you personally believe need improvement?”* Comments by instructors were coded into categories and rank ordered by frequency and percentages for all responses (Table 6). The greatest percentage of responses (19%) involved improvements to training. These suggestions included more trained simulators to help with graduation skill testing with students, refresher courses for instructors, more time in training devoted to preparing instructors for program delivery, less time devoted to in-person training, and more trained instructors. A large percentage of instructors (14%) felt no program changes were needed. The remaining comments focused on updating the curriculum and materials to respond to evolving social safety issues for children in schools and communities, such as more active shooter safety training in schools, updates to current gun safety training, and more age-appropriate curriculum for older students (who get bored with some content and also requested more time for in-depth discussion).

**Table 6:** Instructor Recommendations for Program Improvement

Coded Items	**Ranked Order Responses by Item** **# %**	
Improve training: more simulators, better preparation to teach program, reduce training time, and train more instructors	11	19%
No improvements needed	8	14%
Incorporate more training on active shooter/gun safety	4	7%
Develop more age appropriate material, instructional practices for older students, including more nuanced gender support	4	7%
Evolve program (increased use of technology, social media, internet safety, vaping, etc.) & difficult issues (self-regulation, drugs & alcohol, discrimination)	4	7%
Develop better pacing & organization; sharpen focus on central components	3	5
Enhance training on blocking, other exercises to be more effective	3	5%
Update program materials: coloring book, activity book, parent manual	3	5%
Develop permanent flip charts for instruction and ability to purchase more instructional materials	2	4%
Improve use of multi-media: Session videos for students to follow along in class, short parent videos; use social media platforms to convey messages and send safety updates	2	4%
Increase communication and support from *radKIDS®* headquarters	2	4%
Identify qualified instructors capable of supporting emotional needs of students	2	4%
Develop more on physical self defense	2	4%
Develop advanced skills to stop victimization/bullying (e.g., run/block/tell is not appropriate for all communities or situations)	2	4%
Develop more information to help public understanding of the program	2	4%
Reinforcement of program to support student retention of knowledge & skills	1	2%
Allow non-instructors to assist with large groups	1	2%
Understanding and responding to school requirements	1	2%
TOTAL	57	

## Program Dissemination Recommendations

At the conclusion of the questionnaire, instructors were asked to provide suggestions on their recommendations for broader implementation of *radKIDS®*. Their responses fit under five key recommendations for the program. Reducing training time was the most consistent response, including the recommendation to rely more on technology to replace in-person time. Additionally, instructors saw the need to increase the capacity of the *radKIDS®* organization to expand training opportunities for more instructors and to provide more on-going supports to instructors in the field. A third recommendation was to develop capacity within *radKIDS®* to engage local representatives of the program more strongly in the process of sharing program information with the community. Fourth, many instructors described the need for more community level information about the program to boost awareness of *radKIDS®*. Finally, many instructors in community settings, including parent instructors, expressed the opinion that *radKIDS®* should be in the schools so all community children could benefit from the program. Relatedly, many instructors working in schools felt that *radKIDS®* needed to be integrated into the regular curriculum in elementary school education.

### Discussion: Implications of Findings

Findings from this evaluation helped to confirm the theory of change underpinning radKIDS®, identify program components perceived to positively impact developmental safety among children compared to other available bullying prevention interventions, particularly those predicated on SEL content, and provided recommendations to guide next steps in the program’s development and dissemination. Survey responses confirmed that the comprehensive safety education components of radKIDS® are favorably constructed and delivered with safety planning and behavioral and physical skill training to empower children to become their own agents of personal safety. This focus on child safety and agency, the program’s unique safety education curriculum, and activity-based behavioral skill training delivery distinguishes radKIDS® from other well-established bullying prevention programs such as the Olweus Bullying Prevention Program (Limber et al., 2012; Olweus & Alsaker, 1994; Olweus, 2005), Steps to Respect (Bradshaw, 2015; Brown et al., 2011), the Positive Action Program (Li et.al., 2011), KiVa (Kärnä et al., 2011a; b; Kärnä et al., 2013), Positive Behavioral Supports (Sugai et al., 2001), and Second Step (Gottfredson et al., 2010; Low et al., 2015). radKIDS®, unlike these programs, delivers safety education training that addresses nationally recommended mechanisms for effectively protecting children from victimization and violence, and across the range of settings (school, home, community) that put children at risk of victimization (CDC, 2012; Finkelhor et al., 2014; NCMEC; 1999).

The comprehensive content of radKIDS® addresses multiple types of trauma-inducing victimization that children experience. Many children from minority, rural, or low-income backgrounds are exposed to more diverse and frequent forms of victimization across multiple sources (Gómez et al., 2004; Gallardo-Cooper et.al., 2014; USDHS, 2013). Many children come from environments with high crime and social disorder (Gómez et al., 2004), families experiencing high rates of poverty, domestic violence, substance abuse, or child maltreatment and neglect (USDHS, 2013), and attend schools with greater rates of bullying and inter-personal violence (USED, 2016). Hispanic youth frequently experience greater status insecurity in schools and communities due to ethnic bullying, anti-immigrant and deportation threats, and complex intergenerational disruptions within families (Gallardo-Cooper et al., 2014; Fontes, 2002; Fontes & Plummer, 2010; Méndez, 2006). These multiple, cumulative forms of child violence and victimization are termed ‘poly-victimization.’ Research has shown that such exposure to multiple and different forms of victimization results in more significant trauma than repeat victimizations from single sources (Finkelhor et al., 2007; Turner et al., 2010; Andrews et al., 2015; Breslau et al., 2004; Hatch & Dohrenwend, 2007; Kilpatrick et al., 2003; Buka et al., 2001; Crouch et al., 2000; Schwab-Stone et al., 1995). Most school-based interventions fail to address these systemic violence prevention needs among children and neglect the primacy of personal safety or the tools necessary for fostering this resilience. School-based programs are focused more narrowly on bullying prevention, gains in positive behavior, or the properties of Social Emotional Learning, and fail to address the full context of victimization that vulnerable and social-economically disadvantaged children experience. radKIDS® represents the theoretical foundations and functional empowerment design to bridge this gap in elementary school child safety education.

One of the most valuable contributions of this evaluation was parsing out instructor ratings on the impact of the program related to developmental learning in the targeted safety education components of the program. These impacts included helping children identify unwanted touching (an initial step in preventing sexual abuse), developing effective skills for asking for help and protection when needed, and recognizing and resisting abductions. Child safety items, which we rank ordered according to instructor ratings of effectiveness, also aligned with student survey results from a previous evaluation. Based on these two evaluations, the impact of *radKIDS®* in helping children recognize and report sexual abuse behaviors early in their lives seemed to be a particular strength of the program.

Findings of this evaluation on perceptions of program effectiveness on child outcomes also conform to those of a previous study on *radKIDS®.* In an evaluation of the program’s impact on child safety knowledge, attitudes and behavior, pre–post program surveys were conducted with 270 8- to 12-year-old students in Utah (Johnson-Shelton et al., n.d.; Slater, 2005). Results indicated positive improvements in the ability of children to a) recognize and avoid sexual assault, b) understand the importance of nonviolent responses to bullying behaviors, c) know how to use appropriate resources for avoiding or resisting victimization and deceit, d) develop resiliency by learning to reject victimizing behavior of others as not one’s fault, and e) develop safety behaviors and plans at home. Students reported a 72% increase in willingness to tell a trusted adult about inappropriate touching, a 59% increase in recognizing hitting as not the most effective way to stop a bully, a 31% increase on intention to call 911 if someone threatened harm, and a 30% increase in recognizing that deceit used to inflict harm was not the student’s fault. While our present study may indicate potential bias, the strength of the instructor survey approach was that it capitalized on the use of informants who could uniquely and quickly provide feedback on indicators of the theoretical foundation of the program and service delivery features of the program model. This approach served the purpose of providing preliminary indicators on the research foundation of the program. The evaluation also was intended to inform formative program improvements, which will allow more rigorous evaluations of program efficacy and engagement of multiple informants in future studies. One surprising finding in this evaluation was how differently instructors rated the impact of *radKIDS®* on safety skills in comparison to competencies on Social Emotion Learning (SEL) items. This finding is helpful in differentiating the potential safety skill impacts of *radKIDS®* compared to other bullying prevention programs focused on strengthening early child social and emotional competencies. The SEL items still ranked high in instructor ratings, but as a group, were secondary to safety skills. In effect, *radKIDS®* may provide a potential foundation in safety skill development that can be subsequently enriched by complimentary SEL-focused child development programs. *radKIDS®* is not a replacement for programs targeting SEL outcomes generally. It is complementary. It seems that *radKIDS®* has a very specific and unique focus on child personal safety and victimization prevention, and is more effective in these domains.

Qualitative questions in the survey also provided an efficient means of gathering open-ended responses from instructors both on the impacts of the program and needs for improvement. Responses to these questions also aligned with quantitative ratings of program impacts while also yielding new insights into the child safety development processes on which the program is predicated. While other bullying prevention programs focus significantly on preventing interpersonal violence or aversive child behavior, *radKIDS®* instructors reported a program model that emphasizes child confidence building, generally, and specifically around maintaining personal safety. Related developmental strengths of the program included empowering children to take personal responsibility for establishing their own safety, instilling child self-agency for assuming control for their own safety instead of relying on adults, the strength of the three inter-locking *radKIDS®* rules in providing children a coherent social framework in establishing safe peer relationships in school and community settings, and the individualized safety planning and skills children learn in the program to protect themselves from harm. These interconnected results showed promise for ameliorating childhood risks for diverse forms of victimization and the associated trauma these adverse experiences represent developmentally.

Recommendations for program improvement related to issues in scaling up the program for broader dissemination included (1) refining the training via technology to boost the capacity to train more instructors and enhance behavioral and physical skill competence, (2) creating greater organizational capacity to communicate with schools and communities about the program and support instructional practice, (3) updating and strengthening components of the program in areas of peer and cyber bullying and greater dialoguing among older youth, and (4) simply growing the program to be more broadly implemented in schools and communities. Instructor ratings also indicated a need for more time dedicated for skill acquisition in their training as opposed to seat time. This included more adequate training and support in knowing how to work effectively with parents, cover all material in the intended timeframe, and garner reinforcement for the program from school administrators and staff.

This evaluation survey was internally consistent in demonstrating a) the capacity of *radKIDS®* to increase child safety, and b) acceptance of the program’s value among diverse end users and stakeholders. Perceived program impacts are responsive to national guidelines for safety education and violence prevention programs and are increasingly important nationally as gun violence and other forms of victimization have increasingly entered our country’s social and political narrative.

### Study Limitations

In this evaluation, instructors provided feedback on behalf of themselves, parents, community members, and students. Although this survey approach was an efficient, low-cost method that took advantage of the knowledge, experience, observations, and interactions of program instructors, the method was also inherently vulnerable to bias. For instance, in terms of the reliability of self-report, instructors who responded to the survey were drawn from a purposeful sample of more experienced and engaged instructors who were currently teaching the program at the time of assessment. These participants could reflect a higher level of commitment to the program compared to uninvited instructors or non-responders to the survey who were slightly less experienced in the program, but similar in other characteristics to responders. Relatedly, many instructors who teach *radKIDS®* do so voluntarily and are not paid. It is possible that survey participants could inaccurately attribute positive program impacts for children due either to their inherent belief in the program, or the need to self-justify their personal investments in the program. Additionally, instructors may tend to have reported perceptions by parents, community members, or students that aligned with their own views and ignored, consciously or not, those beliefs and values that differed from their own. Yet, the approach using experienced program instructors as key informants served the purpose of providing preliminary indicators on the research foundation of the program. Additionally, the evaluation was intended to provide formative information to guide program improvements. These contributions were necessary to continue program development with more rigorous evaluations on the program’s efficacy and engage multiple informants with less potential bias in further program evaluations.

## Conclusion

Our evaluation approach, a partnership between the program’s developer and researchers, took advantage of the well-established instructor pool in the program (and the experience base they represented) to describe their own impressions of the program as well as those of other key stakeholders—students, school administrators and teaching staff, parents, and community members. This survey design and approach are relevant to other programs that have established critical engagement over time, and a related pool of experienced interventionists, but lack rigorous experimental evaluations to demonstrate evidence of program efficacy. This evaluation also demonstrates the value of capturing performance measures of a program, soliciting informant feedback in a structured but open-ended format to analyze assumptions about a program model, related impacts, and user group recommendations for program improvements. The approach and findings of this evaluation are useful in supporting a next phase of more rigorous program evaluation and refinement in support of broader dissemination. As such, this evaluation provides a helpful illustration of survey use for advancing the development and scientific credibility of other programs formed in the trenches of community and school practice.

## Supplementary Information

Below is the link to the electronic supplementary material.

Supplementary file1 (DOCX 56 KB)

## Data Availability

Raw data were generated at Oregon Research Institute. Derived data supporting the findings of this study are available from the corresponding author DJS on request. Anonymous survey data collected for this pilot study are available at OSF: https://osf.io/5t43a/?view_only=5dd61aef66cd417ebcdd5a0e24116556
