# Supplementary material for: Program Evaluation of the radKIDS® Youth Personal Empowerment Safety Education Program
Source: J Child Adolesc Trauma. 2024 Mar 15;17(3):831–48. doi: 10.1007/s40653-024-00618-5 (PMC11413255; doi:10.1007/s40653-024-00618-5)
Supplement: Supplementary file 1 — Supplementary file1 (DOCX 56 KB) [file 40653_2024_618_MOESM1_ESM.docx]

**SUPPLEMENTAL MATERIALS**

Program Evaluation of the *radKIDS®* Youth Personal Empowerment Safety Education Program

Journal of Child & Adolescent Trauma

**Appendix A**

**Most Important Aspect of *radKIDS®* Identified by Instructors:**

**Qualitative Responses**

| **Confidence** |
| --- |
| One of the many positive things that I have observed from the program is that it helps students to feel confident in themselves. Self-confidence is key when students are faced with danger. |
| Students have the tools to use to stay safe and understand their incredible value as a person; these new founded attributes create a confident self-esteem that guards the student from becoming a victim, even when harmed. These students will fight back and not be victims |
| To teach them to protect themselves and not hurt others but to stop them gives the children a confidence about themselves. For those questions I gave only a slightly agree, it is because those are hard to judge for 4-year-olds. This program is phenomenal! |
| The self-confidence achieved through eight hours dedicated to them, this is the beauty and effectiveness of *radKIDS®* in my opinion. The most positive impact of *radKIDS®* is self-confidence and empowerment. |
| Self-confidence increases as the children go through the program |
| The foundational principal of empowering children to be responsible for their personal safety. The confidence graduates of the program show is strong in comparison to the student on day one. |
| I feel they are more confident, able to stand up & speak out not only for themselves but for their fellow classmates if needed. |
| Self-confidence, safety awareness, pro-active behavior, and greater ability to communicate |
| I feel it gives them confidence to be able to handle emergency situations that may come up in their lives. It also gives them so many tools to be able to resist aggression defensively in case of a possible abduction or assault. |
| Build confidence in protecting themselves. Understanding that it's ok to say "no" to an adult. |
| Helping the kids feel empowered to protect themselves and to not be scared to speak up and tell. |
| I have noticed a large increase in confidence of children who complete the *radKIDS®* program. I love that these children feel empowered and secure and that they always have a plan before they go somewhere. |
| **Empowerment** |
| Helping empower children to find their "voice" to yell loud, hit hard, and run fast to safety. |
| Children are empowered. *radKIDS®* helps children gain their voice when feeling sad, scared or confused. They are taught clear tools to stop a predator, signs to look for, ways to keep themselves safe and to tell a trusted adult if someone has made them feel. |
| Empowerment, reducing fear through development and practice of realistic plans of action, positive relationship with adults. |
| I believe all of the instruction is beneficial in their young lives. I love that the children can return each year to reinforce the skills they've learned and empower them into the future. I'm a true believer in the program and have been teaching it over 7 years. |
| The self-empowerment it teaches the children. They become more secure in their ability to help themselves and to handle a situation. It also helps teach them to remain calm in a crisis and how to call 911. |
| Empowerment and personal success when utilizing the tools and options we have taught them. |
| Knowing what being empowered to protect themselves means is everything. They know they have choices. They learn how to use their tools to take care of themselves. |
| Empowering them to think for themselves rather than wait for an adult to think for them/tell them what they should do |
| Empowerment to take action to keep themselves or others safe, with less concern about “disobeying” adults (i.e. permission). This is applicable in settings where bullying occurs and adults have useless “solutions” as well as situations where the adult is trying to trick or hurt a child |
| The empowerment that comes with their ability to have boundaries and personal rules that are their very own. |
| Our children find their own power through this empowerment program. In my view supporting two school systems K-5 for the last seven years, this program is a first step in prevention of human trafficking and a prevention/intervention program for child abuse. |
| Empowerment regarding making their own decisions. While children instinctively respond according to familial rules and mores, seeing that light bulb go on when they "get it" that they CAN say no to an adult in specific situations. |
| Just what it says. SELF EMPOWERMENT. Kids get anchored in how special they are. How special others are. How to take care of themselves in regard to personal safety in a variety of settings. As we say a lot, "*RADKIDS®* just know more." And with that more comes confidence in their abilities and self-worth. |
| **Self-Agency** |
| Understanding that they have responsibility and ability to keep themselves from harm. |
| Allowing the students to protect themselves in a positive way while using strong words and powerful movements |
| *radKIDS®* training teaches pre-school and school aged children to recognize, avoid and resist harm and danger in their lives. As a parent I am passionate about this because the fact is we as parents cannot be with our children every minute and we cannot think for them in all situations. *radKIDS®* equips them with skills to act over their fear to avoid harm. I believe if every elementary school aged child received *radKIDS®* the incidence of bullying and cyber-bullying would be drastically reduced. |
| **Plans & Skills** |
| Physicals skills to help develop confidence and capability |
| Drills seem to be the most effective form to train the children. |
| The combination of teaching the physical and educational is a very good tool to keep their interest and make them work through. The kids relate to the material and have been very involved. The teachers love to help and also love the approach. I talk to them later after the classes and they always bring up something that they recalled or that they wanted to share, it’s a great medium. I did not get that from DARE. |
| The physical skills *radKIDS®* learn are a positive impact because they learn a skill and have fun while doing it. The drills also require them to think critically during a stressful situation. |
| When we teach bullying it gives the kids a way to recognize it and how to handle it. Often times kids are just talked to about bullying they never really have ways to handle it. With *radKIDS®* they are taught ways to stop it. |
| The physical drills bring everything together we teach. It creates great follow-up dialogue when you do the "recap" portion of what they learned the previous day. Most kids connect by visually learning and I believe the *radKIDS®* program does an incredible job with the visual aspect of the learning. |
| The most important is using the stance and their voice for self-defense. the 3 rules for *radKIDS®* empowers them to have more confidence in making decisions in an emergency. Making their own decision of which safe zone to use. Password safety is very popular as well. Knowing what is going to be asked by the operator when calling 911. and the drills are also very powerful in having a plan. |
| They have OPTIONS for being safe and have the power to make choices that can lead to being safe. |
| **Stopped Victimization** |
| A student at our school used the learned skills to avoid a possible abduction. |
| How to recognize and report sexual misconduct. Our classes over the years have several reports of empowered children involved in situations that have stepped forward and stopped it. |
| We have had one student disclose abuse. |
| **Voice** |
| Using their voice, creating awareness of dangerous situations, improving self-esteem and gaining confidence. |
| I love that this teaches the kids that they have the right to stand up to anyone trying to hurt them. |
| Confidence to tell someone no if they are not comfortable with them |
| Giving the kids tools to fight back and get away from unwanted situations, I've seen them come in quiet and reserved to by the end of the course, having confidence in themselves enough that they hold their heads higher and feel more empowered. |
| **3 *radKIDS®* Principles** |
| The three principals and the 9-1-1 practice |
| I think the "3 rules" has a very positive effect on the kids, with perhaps the third most. They seem to be quite interested in them. |
| The 3 *radKIDS®* Principles |
| I think relating concepts back to the 3 things all *radKIDS®* know is key. If kids believe in those 3 concepts, and you can show them how something is related to something they believe in, the rest is easy. I believe *radKIDS®* teaches all of the above extreme |
| The 3 *radKIDS®* rules, they can say them and apply them in all that they do at school and at home. |
| Learning and adhering to the three *radKIDS®* rules. |
| The 3 *radKIDS®* rules! Important to know at any age. |
| I love the 3 things all *radKIDS®* know. At any age, no matter the situation, you can use some aspect of the 3 rules. Also, I love teaching about good people and bad people. Helping kids understand that it’s not “stranger danger” anymore, they can evaluate any situation |
| Just knowing that no one has the right to hurt them and that they can come up with their own rad kids plans have a positive impact on the students. |
| *RadKIDS®* rules! They take them to heart. |
| The 3 *radKIDS®* rules give children an understanding of their worth and their importance. Once they internalize those then they can have the confidence to fight the bad guy, stick up for themselves, and keep telling until they are heard. |
| The three rules in their daily lives. |
| Students using *radKIDS®* language, "you don't have the right to .....". Also, we had a student at Disney who successful identified a person and got away. |
| The 3 *radKIDS®* rules; Sam’s Secret; all drills; the bullying approach |
| **Saying "No" to an Adult** |
| Knowing that it is perfectly acceptable to say NO and to use what they have learned to protect themselves. |
| The fact that the kids can tell an adult "no". Having a background as a detective, I unfortunately got to see what happens when adults (family, stranger) victimized children. Letting them know that no one has the right to hurt them, sends a very string me |
| The physical part is important. However, for the children to know that it is alright to say "NO" to an adult and to ask for help has been a very positive thing. |
| I believe *radKIDS®* empowers children to be able to say no to an adult if they feel uncomfortable. |
| Recognizing that adults are not always doing the right thing, and that they may be trying to trick them. Also, knowing how to defend/flee a bad stranger. |
| Permission to resist an adult rather than be compliant to any adult they encounter. |
| **Identifying Danger** |
| Knowledge of tricks and how to tell if someone is doing something they should not to hurt or trick them. |
| Being able to recognize their feelings as a means to learn more about a situation, avoid danger, or seek for help. They have an understanding of their importance and are comfortable yelling in a crowd, calling 9-1-1 or grabbing on to an adult they trust |
| Identifying risk and creating a plan to avoid violence or danger |
| To know how to define unwanted touching. Also, how to be safe in a community. I really like the good people, bad people take rather than stranger danger. |
| Realizing that they have they choice to say "no" to someone, even if it is a relative or a friend. Confidence in identifying unsafe situations, enacting a plan and identifying safe people who *radKIDS®* can go to for help.  Focusing not only on bullying behaviors the radKIDS may have seen or experienced, but also looking at their own behavior to see how it affects others. Gaining confidence through physical skills training. Using the Drills on the Fly is a fun and exciting way to practice lessons and work out plans. The kids LOVE the drills! |
| **Self-Worth** |
| Believe in themselves, they are special and no one has the right to hurt them. |
| Students learn that they have value as a person. They learn rules about how others should treat them and how they should treat others that can be drawn upon in future situations |
| The self-worth *radKIDS®* teaches gives children the confidence to say no when needed. It helps them to create their own boundaries. |
| The most positive impact that I have witnessed is their understanding of their own worth. Once they recognize this and understand it, they begin to act with more confidence and power. Knowledge of their personal value and recognition of the value of others. |
| Believing that they are important and we care about them. If they believe in themselves and their worth they will take care of themselves and others around them. |
| **Child–Adult Connection & Communication** |
| The rapport that this program builds between the children and the instructors teaching and empowering the children who attend the course. They never forget their *radKIDS®* instructor. Most do not forget the life skills they learn in *radKIDS® ®*. It is awesome to see the children the first day of the Rad Kids program and then compare it to the last day of the program. The change is priceless and gives me the goose bumps every time. Every child should take this valuable program. It is not only that they learn a lot, but it is also fun and full of physical activity which is important for children to have. |
| Our students have learned that they have the right to say no to unwanted touches and that there are many people they can talk to if they have a problem. |
| Bullying and the knowledge they can get help from adults. They have learned to learned to keep asking even if they don’t get help the first time. |
| **Reduced Aggression/Order** |
| The hitting in my classroom has gone down tremendously after teaching the *radKIDS®* curriculum. |
| The structure and subsequent orderliness of the class, including the coordination and cooperation among the students, while learning personal safety practices and techniques. |
| **Other** |
| Being good role model and mentorship |
| I think the fact that we are talking about issues that parents and teachers either don't know how to talk to there kids about anymore or don't have time for in school because of all the other demands in the lesson plans. The info that we send home of what we talk about that day allows parents to talk to their kids at the dinner table that night and not only opens the door to conversations but also helps brings more safety and awareness to both inside and outside of the home. |
| Awareness of their actions on others |
| The children going against the red man, realizing they have the strength and skills to do so. |
| When we involve parents, students internalize the content and drills. |
| Note: responses often included multiple aspects of *radKIDS®* deemed by instructors to positively impact child development and were coded under multiple categories. Also, simply phrased comments like "confidence" are not posted in this table but were included in the coded analysis. |

**Appendix B**

**Most Important Aspect of *radKIDS®* Identified by Stakeholders**

**as Reported by Instructors: Qualitative Responses**

| **Importance of RK to School Administrators** | **Importance of RK to classroom teachers** | **Importance of RK to students** | **Importance of RK to parents** | **Importance of RK to community** |
| --- | --- | --- | --- | --- |
| Bullying, school and community safety | Bullying, school safety, self-empowerment and confidence | Fun, skills, drills | Resisting abduction and sexual predators, overall safety, skills | Community safety, resisting abduction and sexual predators |
| This program helps differentiate our school from others since we are the only school in our area to offer this program. | Provides a common safety language for children to use. When teachers are unsure about how to handle certain situations, they ask for assistance/support. | Child empowerment! This program builds students' confidence and teaches them how to recognize and resist danger! | Many parents are afraid to cover such sensitive topics with their children in fear of scaring them. Parents are thrilled with the program because they know that the information is taught in child-friendly ways. They love the pad work and simulation aspect | The community would like to have *radKIDS®* offered at more locations than just our school site in order to reach more children in our community. |
| safety of students | managing classroom behavior | having fun during training | children's safety | children's safety |
|  |  | Personal safety, self confidence | Child safety, child self-confidence, how to teach it |  |
| They get to ensure safety to the school and check a box that a safety program was taught. | Re-enforcement that everyone matters in their classroom and tools to build self-esteem and confidence. | They love the *radKIDS®* moves and that they can fight back whether it maybe words, physical harm or emotion. | Assurance that their child has a plan if there is ever danger and that their child will tell if any harm happens. |  |
| Getting the message out in a positive and meaningful way for students of all ages and their families to protect themselves from danger | Student involvement is high Students retain the information and the physical stances | Students enjoy learning the physical moves and yelling | Parents enjoy the family manual | Community members are thrilled that students are learning to protect themselves from harm and danger |
| Because of our society that we have now, it is imperative that we teach our students to protect themselves. School administrator is totally for it. | It is a big help for behavior management in the classroom. We go over the 3 rules daily and remind students that *radKIDS®* are cool kids and they go from danger to safety. | It gives them a confidence about themselves and that they are important, each and every one. | They like the extra protection the program provides in teaching their students to stay safe. | This program helps to instruct our children, our future, to treat others with respect but when others try to hurt them, they have the right to protect themselves. |
| Children thrive in environments where they feel safe to be themselves and not be harmed, intimidated, or verbally-bullied by others. |  |  |  |  |
| The students not only police each other(look out), but they also do so for themselves. It makes it all around easier to manage your day, and not deal with constant behavior issues. | Simply reminding students on how *radKIDS®* behave works very well. They remember that *radKIDS®* know more. | Their confidence goes through the roof. And giving them the power to say no and protect themselves spikes that confidence exponentially. | The students go home to teach parents what they've learned. Then the use it in their daily lives. Every lesson learned can potentially save their child's life. | The lessons can make it easier to manage "problem" behavior. Because students learn what is acceptable behavior and what is not acceptable. |
| The students have a greater sense of pride and they feel safer in a school environment after taking the classes. They are able to explain to administrators what the problem is and ask them for help. | The program teaches respect for classmates, and how to resist bullying which makes a teacher job a little easier. | Empowering a child always helps them feel and be a little bit safer. | The program teaches children some safety that parents sometimes don’t think to tell their children. | Our program has had a huge impact on our community in many ways. Children feel they are empowered to participate and contribute to local activities without feeling afraid. |
| Most schools in our area have never heard of the program. |  | Self-defense moves and dog safety | Child safety | Child safety |
| School administrators are fearful of lawsuits and have stated they like the program but fear the children will use it to harm each other. | School administrators are fearful of lawsuits and have stated they like the program but fear the children will use it to harm each other. | School administrators are fearful of lawsuits and have stated they like the program but fear the children will use it to harm each other. | Parents love the program and have expressed how impressed they are with their child. Community centers and churches are the organizations most willing to have this type of training. | Community centers and churches are the organizations most willing to have this type of training. |
| Tools to handle bullying. Student self-control | I'm not sure how/if teachers use what we teach and review. We ask teachers to sit in on classes so they can see what we are teaching. Some information/suggestions on how to use the principles might me helpful. (an area for improvement) | Self-confidence; self-image. | Student ability to protect themselves; Sam's secret should be having a big effect. We should follow up with parents to see what they've observed | Child safety and response to dangerous situations. But we need to get more information out re what we teach. |
| Our school district is not involved in *radKIDS® ®*. They do however allow me to use the school if I wish to teach it as an after school activity through the girl or boy scouts in which I have done in the past. |  | Students enjoy the program so much they have returned every year until their 13th birthday. I always have positive comments on my evaluation forms. | Parents are very supportive of the program and have put all positive comments on the evaluation forms provided. | The borough has always been very supportive of the program within our police department and has funded me every year to assist with needs for teaching, materials, parent and child handouts and membership dues. |
|  |  |  | Parents who have attended the training with their child have very positive feelings about the program as a whole. Several children have returned yearly to receive the training again. |  |
| Implementing core values and *RadKIDS®* rules | Understanding every kid is unique and special - every kid is a RadKID | Learn the importance of being safe, boosting their self-confidence and self-esteem that he / she can handle unsafe situations | Empowering their children and helping their children stay safe | Increasing community awareness of helping kids be safe in all aspects of life - safer kids, safer community |
|  |  | kids love learning new things and being able to show they can do it | gives parents confidence in that children will react |  |
| Curriculum based information | Behavior management | Self-value | Safety that can be used when their children are away from supervision | Crime Prevention |
| Growth in confidence and ability to cite *radKIDS®* principals when addressing future student behavior issues. | Growth in confidence and ability to site *radKIDS®* principals when addressing future student behavior issues. | Had fun learning new skills. | Interaction with law enforcement in positive environment. Reinforcing safety topics with student. | Safer kids. |
| The self-defense techniques are effective and even the most uncoordinated and uninterested kid has been found to become involved and enjoy. the teachers love it and say that it is instruction that is often overlooked and they wished that they had it when they were younger. | They like the relevant and no holds barred approach as well as the actual physical portion and working towards getting away and telling. | its empowering and they enjoy the fact that they get to practice new things and be loud in school and the police officer is telling them that he wants to get in trouble because everyone thought they were too loud. They beg for the time and want to keep go | same as above the parents like that the kids are having fun but also reinforcing what they have been teaching or been unsure of how to. It shows support and community involvement. | Same your taking a vested interest in the kids and that is always a win. |
| The biggest topic we cover, as far as the school is concerned, is bullying. | Again the bullying part of the program. | Empowering them, letting them know that they have control over themselves, that they can tell an adult "no", and in the end they can win against the bad guy. | my own daughter went through *radKIDS®* several times, and I can personally say that when it came time for the dynamic simulation, I was very surprised. I was confident that if she were ever put into a situation where someone was trying to hurt her, she could defend herself | Keeping our kids safe. from bullies, predators, and just bad people in general. having less victims. |
| School administrators loved the program when we were able to teach it at the school. The district started charging us to teach the class, so we stopped doing them at the schools. |  |  |  |  |
| For both school administrators and teachers, the most important aspect was for students to be able to recognize abusive behavior directed towards them (bullying, inappropriate touching, etc...), how to resist/avoid it, and how to report the behavior |  | The students always love the physical portions of the program, whether it's the drills on the fly or the pad work for striking. Anything where the kids are learning to verbalize and use their skills in context is most important to them. | Parents want the kids to learn how to advocate for themselves, and know their kids can be safe without mom or dad present. The rule of context is also important to parents, so kids are using their physical skills under the correct circumstances. | I think the community partners that support these programs, such as PTO's and Boys & Girls Clubs, want to know they are providing the support system necessary to deliver the program. These groups need to understand the goals of *radKIDS® ®* |
|  |  | The drills have been the most important to the children. | The parents were glad we reads the "Sam's Secret" book and addressed sexual abuse. The discussion on internet safety is a huge concern and valuable to the program. | The community is excited about the program and I have had a waiting list since November 2017. I've taught 3 classes in less than a year. One 5-7 age group and two 8-12 age group. |
| School administrator like the safety portion of the class, | Confidence builder for the kids, observation of behavior | fun environment of learning valuable skills, | parents feel it helps their children to recognize danger |  |
|  |  | Students learn many life skills and the rapport that it builds with law enforcement as their instructors is priceless. I have had students who were in fifth grade attend my class and then when they are seniors and some in college remember me and the program. | I receive nothing but positive feedback from the parents whose child has attended my *radKIDS®* Program. They tell me they are so grateful for everything that I have taught their child. It is a great feeling to observe the parents reaction…. | This has been very positive in the communities we serve. The word gets around about the program and I receive requests from parents in the community to have their children attend my programs. It is so popular that at times I have to refer them to other *radKIDS®* programs in other communities. |
| Bringing everything back down to the basics and the simple principal that the kids themselves are the most important person in the building. | Allowing great teaching points. | Talking about issues that parents and teachers sometimes forget to talk about. For example the dog defense and or the safety meeting spots. | Allowing talking points and terms used in the good touch, bad touch and unwanted touch is a great way to put a difficult conversation to have with their kids. | Allowing the PD to bridge the gap with the community is always important. |
| The ability to help empower the child to make their own decisions. | The interpersonal relationships between different groups of students. | Ability to become their own person. | The child being able to protect themselves when the parents aren't there to do so |  |
| Not sure about the school administrators, that was dealt with above me. | The teachers were looking forward to the classes taking place. They felt that the bullying and "rules" were important and were able to incorporate that into their daily routine. | Felt that they had a voice now and that they didn't just have to accept it. They also knew that they could speak up and not always have to go "tattle" to the adult, unless the steps they learned did not work. | Felt that their children had more confidence and thought they were more safe if they got into a situation where there was no adult present. | That children are learning to respect themselves and others a little more with the confidence that they get in the class. Also that they are not an easy victim any longer. |
|  |  | Physical skills with accompanying principles for their use. | Education and skills. |  |
|  |  | teaches them to value themselves, to protect themselves, to come up with safety plans and have confidence | Educates them about important topics and gives them a way to bring up certain issues that they maybe would struggle to bring up on their own | Provides a resource, allows the community to build a better relationship with law enforcement |
| Teaching of personal safety to children, safer environment | Students learn self esteem | Learning to value themselves as a person, know that they deserve to be safe | more confidence that their children can defend themselves | Children that know how to remain safe |
| Sexual abuse and predators | Sexual abuse and predators |  |  |  |
| Safety | Safety, bullying | Safety, every student is important, fun, interactive | Safety, bullying, internet/social media, | Safety |
| Good bad touch | Good bad touch | Defense tactics | Defense | Awareness off safety topics |
|  |  | I feel students are more aware of their ability to speak out and say NO! They are more knowledgeable of how to be safe not only at school but also at home and in their community |  |  |
| Students know how to let adults know if someone is bullying them. | Students are able to control their behavior, if someone is bothering them they know what to do. | Students learn how to protect themselves in a positive way. | Gives parents a peace of mind that they have been taught ways to protect themselves and know that they are special and important at home, school and in the community. | Gives students a voice. |
| Sense of community. |  | Self-empowerment | Self confidence |  |
|  |  | Students feel confident, strong, and prepared | Parents feel relieved and grateful that their child can defend themselves. They also notice more confidence in their child. |  |
| I provide the school administrators the information regarding the program and what is written on the website is all I need to show them. They are sold on the program and have no reservations as to keeping the program going at my school. | Helps eliminate bad behavior and students making good choices within the classroom. | Helps eliminate bad behavior (bullying) and students making good choices in the classroom, during recess and at lunch. | Parents like to see the behavior of their students improve at school and at home. They are also very relieved and feel a sense of comfort knowing that their children have learned and practiced many safety issues for the home, school, car, out and about (s | The *radKIDS®* program brings a sense of relieve to the community in knowing that our children and safer from potential harmful people and situations since our kids are now empowered with the knowledge that they learned in class. |
| My administration likes the program, as it helps parents decide to enroll their children in our private school. | Our classroom teachers aren’t involved in the program. | Students become empowered to make choices and they have the ability to say no. | Parents love the program. It helps them feel more secure when they are not with their children. | I don’t know if our community knows much about it. |
| How to handle/deal with and understand what bully situations actually involve. | Not much impact on classroom teachers, but supportive. | Learning to value themselves and understand that no one has the right to hurt them. | Hopefully increased confidence that their student is prepared for any unwanted situation. | Unclear on community involvement outside parents. |
| Bullying Section | Bullying and awareness | Bullying | Bullying, | Bullying |
| Safety of children | Safety of children | Safety of children | Safety of children | Safety of children |
| Bullying | Bullying | Bullying | Bullying | Bullying |
| teaches children how to be safe in school and community | helps the teachers stop bullying and bad touches because the students are empowered to tell | Students learn when it is OK to tell and how to say no to behaviors that they should not have to put up with. | The Family manual is a resource for the family to know what was taught to help reinforce it. | teaches children how to be safe in school and community |
| They see how important it is for their students to learn how to defend themselves and the confidence it gives them. | They see how important it is for their students to learn how to defend themselves and the confidence it gives them. | They see how important and beneficial it is for them to learn how to defend themselves as well as the confidence it gives them. | They see how important it is for their students to learn how to defend themselves and the confidence it gives them. |  |
| This is a program that addresses bullying and cyber-bullying. | *radKIDS®* principles of 1- No one has the right to hurt me 2- I don't have the right to hurt anyone else including myself unless someone tries to hurt me then I can stop them 3-If anyone does hurt me it's not my fault so I can tell | School and home safety as well as out and about safety | bullying prevention | education about safety at school |
|  | Helps kids identify bullying and report it. | Helps kids to have confidence in knowing what to do in certain situations. | Helps teach safety lessons they might have overlooked. | Helps keep everyone safer. |
| Bullying | Bullying | 3 *radKIDS®* rules | Safety out and about, Home and school | Stranger danger, drug safety |
| Child safety and awareness | Child safety | Their safety and knowledge of what to do in unsafe situations | Child safety and parent awareness |  |
| Anti-bullying, self- empowerment | Anti-bullying | Physical resistance skills | Physical resistance skills |  |
| Teaching children they don’t have the right to hurt anyone else. That includes peers, teachers and other faculty members | The classroom teachers in our school need to better understand this program. I don’t feel they grasp how it could benefit their classroom environment | Understanding their self-value and purpose. They learn they can and should stand up for themselves and others even if it is an adult or older child. | The knowledge in *radKIDS®* should improve their home environment in all aspects. However I would say a large majority of parents are unaware of what their kids are learning. | If all would adhere to the philosophy and teachings of *radKIDS®* we would certainly build stronger and safer communities. |
| safety in schools, *radKIDS®* answers the question: What are you doing to help my child be safe at school | helps teachers be on the same page when it comes to bullying and not hurting others. | The self-confidence and skills they gain | Knowing their children are being taught to project themselves and have respect for themselves and others. | raising more aware and involved children equates to better citizens |
| Safety at school (to and from, bullies) | Personal Boundaries, classroom safety, bullying prevention | Personal protection-skills | Abduction prevention skills...knowledge and physical skill....so many moms cry when they come to simulation. | Child safety and well being |
| I'm fortunate, the school fully supports the program. However, we need more staff trained and that's been difficult to get planned. | The teachers are in the classroom when we teach the curriculum, which is great. They are all very supportive of the program. | Some students thinks it's lame. But most seem to take it seriously. | I've only ever received one comment from a parent that her son thought the program was lame. My response to her was, "that really surprises me because he's the one the volunteers with most in class with responses to questions and is very participative in | I only work with the community when the officers reach out for assistance. Which these days is not often. |
| Helping the children make decisions for their own safety. | the difference between tattling and telling. | Understanding their ability to control their safety in many situations. | For the parents that do attend, the ability their child develops to understand their rights to take care of themselves. |  |
| Potential for decreased bullying. Opportunity to build self-esteem. | Potential for decreased bullying. Opportunity to build self-esteem. Options for classroom control (using the 3 foundational principles, and *radKIDS®* rules). Increased focus and safer place created for better learning. | Increased self-esteem, understanding of self-worth. Opportunity to defend self against threat, harm and/or violence. Because this class is all about them, and taught on an individual level, it naturally creates a safe place and way to learn. This is "f | Opportunity to learn life skills in the school setting. Generally free. Increased self-esteem. Positive, fun way to learn safety skills which they may not know how to discuss otherwise. Avenue for parents to volunteer in the schools and assist. | Opportunity to break the cycle of violence that exists when children learn behaviors from a 'criminal natured' parent. Learned behaviors enhance the community with children who care about themselves AND others. Creates more positive, empowered, productive citizens |
|  |  |  | "The 3 Things All *radKIDS®* Know," safety plans, bullying curriculum, private parts, strangers | "The 3 Things All *radKIDS®* Know," safety plans, bullying curriculum, private parts, strangers |
| Bully prevention | Bully prevention | Confidence they can be safe on their own | Sexual Assault Prevention |  |
| Opportunities for additional enrichment after school. | Confidence building. | Self-confidence. Learning they are in charge of their safety and can stop anyone from hurting them. | ALL OF IT. | Helping make children, and in turn, our community safer. |
| Leadership and better choices. | More respect | Helps to build self-confidence and knowledge as to what to do if/when bullied. | Safety and self-confidence. |  |
| Unfortunately, I feel that our school has allowed us to bring the program to the school but it seems to be an annoyance or something that they just let us do, but there is no support to make it a part of the school community. | The teachers bring their classed down for their time to participate in the program, but they have no other interest in the program. The teachers see it as a good thing, but also a big thing that gets in the way of their teaching. | Allowing them to talk ask questions and share experiences. | Finding a way to really get the parents more involved. Many parents sign the release forms to allow their students to participate, but they do not really know about the program or do much with it after their students class is finished being taught. | Some people in our community know about the program, but nothing more has been done to bring the program to the community, |
| Decrease bullying. | Improved respect of children for each other. | Improved self-worth and knowledge of how to keep themselves safe. | Helping their kids stay safe. |  |
| bullying, self-confidence, | school culture of anti-bullying and abuse | relationships with adults who care about them enough to teach them such important life skills | self defense | Anti-abuse |
| The ability to recognize the worth of themselves as well as others they interact with. | The ability to communicate with each other and with the teachers. Also, the ability to distinguish between problems that need a trusted adult and those they can try to work out on their own. | Knowing they are special. Knowing they can say NO to an adult in the right circumstances. The ability to defend themselves should the need arise. | Knowing their kids are learning to be aware of safety issues and learning how to proactively mitigate those risk factors. | Kids who are more aware are safer. They know how to call attention to safety hazards or dangerous people, protect themselves, and make their living area safer. This helps the entire community to watch out for each other and be safer. |
| Teaching respect for self and others. They liked all the safety instruction and lessons on bullying. | They liked the lessons on tattling vs. telling and instructions on safety. | They loved learning how to protect themselves and how to make a plan in case of danger or disaster. | They loved the ideas of passwords and the students being protected from kidnapping and harm. Some parents used the manuals given to talk through safety ideas and how to deal with fire and disasters with their students at home. | Our community sponsors *RadKIDS®* because a community with kids who are keeping themselves from harm and protecting those around them from dangerous situations is a community with a purpose and a unifying goal. We work together to see our children are safe. |
| Safety | Safety | Self confidence | Safety | Safety |
| Support and involvement | Support and involvement | Involvement | Support and involvement | Support and involvement |
| Honestly, for the administration it just makes them look like the care about students safety for parents but they really don't know much about *radKIDS®* and even with invites to come and participate, it's not high on their to-do list.  Wish they'd get more | Teachers care about their students’ safety but are not involved enough when we come to the classroom to teach. Most of the time they're doing prep work during the course. Most see it as either an inconvenience or an extra free period to do prep work. | *radKIDS®* is most beneficial to the students; this is where we see kids confidence grow! This is where it's most rewarding to us instructors because during the course and towards the end, kids gain a trust with us and their confidence and personalities really develop | Peace of mind knowing their child can think through any dangerous scenario and plotting a plan to stay safe. | Keeping a watchful eye and setting up safe zones for kids to run to when they need help. |
| *RadKIDS®* rules assist them in helping keep them safe and informed. | All levels of safety | They like hands on teaching | Hands on and constantly reinforcing *radKIDS®* rules | Awareness and preparation for safety situations |
| The password is recognized by others as an invaluable tool for families to use. A lot of parents and teachers become interested because of the physical resistance to prevent an abduction, but as their child progresses through the program, they identify v |  |  | The password is recognized by others as an invaluable tool for families to use. A lot of parents and teachers become interested because of the physical resistance to prevent an abduction, but as their child progresses through the program, they identify v |  |
|  | After sitting in a class, I've had teachers thank me SO MUCH for teaching these valuable things to the children. They recognized that *radKIDS®* covers important stuff to help the children grow up and live healthy lives--preventing drug use, abuse, abduction | Some kids are very scared and hesitant at the start of the course. By the end the kids feel so strong, aware, prepared, and confident. Some students still elected to not participate in the simulation, but usually because they didn't want to hurt the person. | Many parents have thanked me for teaching their children. Some have said that it helped to start a conversation at home about safety topics--emergencies, "bad guys", escaping danger. | I've taught for 11 years. The kids I taught as first graders are now Seniors in high school. Some of them still remember me from teaching them *radKIDS® ®*. It made an impact in their lives and in turn will impact the community as they graduate and become adults and parents |
| Anti-bullying and internet safety. Loves the “nobody gets hurt here” instead of “zero tolerance” | Not sure. Very little feedback from classroom teachers. | The drills. By far their favorite. They love the fire safety (under the parachute “smoke”) and anything that involves being chased by a “bad person.” They also enjoy using their skills on Bob and Steve the dummies! | General safety skills for their kids. Not a ton of feedback, but the little I’ve gotten has been positive for the program in general. |  |
| Anti-bullying tactics | Bullying and rule 2 | All of it | The strategies for avoiding abduction and bullying. | Same as parents |
| In my context school administrator means the head of my martial arts school. The aspect of the program most important to him is that it teaches kids context and permission, which is not always a direct component of martial arts training, weirdly enough. | The teachers in my martial arts school absolutely love *radKIDS® ®*. It gives them a well- founded, well-grounded structure in which to teach kids physical and emotional strategies the context of real life situations. Sam’s Secret is a game changer for my mart | They become less fearful and more confident. They understand when they need to use their training and how. They learn that they are in charge and when their safety is at stake they can tell, yell, make a scene, break things, use physical resistance. | Understanding that this is not a stranger danger program and why. Understanding that we help kids make a plan for personal safety in any context when their parent may not be around | A community committed to *radKIDS®* will see a drop in all forms of bullying, with the attendant side effects of that decline. Many other aspects but that is a huge community concern |
| Provides a foundation for developing the culture/climate of the school. | It would provide a foundation for classroom rules if used. | Gives students the foundation skills and permission to protect themselves. | Gives peace of mind that kids have the skills to avoid or escape a dangerous situation. | An educated community is a safer community. |
| The language students practice and drills | Would like to see classroom teachers Trained, since they are with the students more and can respond quicker to an event (bullying on playground or who is picking them up) | I think all are crucial- our students are not riding busses or walking to school, but we set it up as field trips or walking to friends’ homes. | Would like to see more parents come to our simulations/stations. | Getting the word out about how important this is. |
|  |  | Having plans for different situations | Reinforcing or introducing safety rules | Gives the community a safe place to send their kids to prepare them to live in the community safety |
| Bully prevention | Bully prevention and resistance | Staying safe while “Alone” (strangers, out and about, walking to school, Etc.) | Abduction and sexual Assault prevention | Abduction and sexual Assault prevention |
| We focus on social and emotional growth as a school. Our kids will still continue to receive this program in k- 3rd grade. | Because they are certified trainers, they can easily implement the program on a daily basis. | Students enjoy learning at this age. They are very engaged and they remember the skills and drills. | As parents they love the program because it teaches students how to defend themselves away from home and at home. Parents are asked to follow our program and continue to teach at home by using their manuals and homework assignments given by the teachers. | The community is beginning to learn more about the program as we continue to teach. I think the program is positively received as we continue to progress. |
| None reported | Better ways to avoid conflict besides resorting to violence | Getting a bully to stop picking on them and how to be safe in the community | Their children learning how to stand up for themselves and say no | None reported |
| Bullying | Bullying | Empowerment | Children’s safety - all of them | Children Safety - all of them |
| All around safety component with personal safety in the mix. | The all-around safety focus and discussion around respect. | The students love simulation and the ability to use the skills they learned. | The information on sexual abuse prevention and the physical self-defense. | Creating awareness around child sexual abuse. We have to be careful that the message of 100% perpetrator responsibility doesn't get lost. |
| Getting to think on their feet | Improve school climate and culture | Confident and know what to do. | Ensure their children know what to do in a potentially dangerous situation. | Safer communities and lower the cost of social services needs and costs. |
|  |  | Rules, Stance and skills. Sam's Secret is very impressive to them, when it is read we could hear a pin drop in the class. they are so attentive. | Stance, password and skills taught. | Rules are clear to children. Stance and skills are practiced. They learn to use their voice and are able to speak up. Also they like Sam's Secret. |
| The rules , the tricks, good people bad people and the tactical skills | Counselors Sam’s Secret | Rules ,Tactical skills , drills on fly, | EVERYTHING!!!! | Everything!!! |
| A good overall safety program that encompasses more than just bullying and physical resistance. Including home, school, bike safety etc.  Shows effective means of identifying, avoiding, and helping in situations of bullying. However, I have found that t | Gaining confidence and self-worth, as well as identifying unsafe situations. Learning to think and plan a solution to an unsafe situation. Gaining confidence in going to get help. Assessing their own behavior to see if it is acceptable | Having a safe and fun environment to ask questions, get feedback, and make mistakes. Knowing that if they don't make the best decision in a "trick" drill, we can talk about it and give it another go. Watching the kids become more supportive of each other | Situational awareness. Having a plan. Giving parents a place to start having the discussions and make a family plan. What will we do if we get separated at the mall? Having everyone know the same plan. Where will we all meet if there is a fire? | As the program becomes more widespread, hopefully the community becomes more supportive of everyone within it. Listening to children, watching out for unsafe behaviors or "bad" people. Knowing what to do and what resources are available if a *radKIDS®* asks |
| *RadKIDS®* graduates contribute to a calmer atmosphere in the school. | As above, the graduates are calmer in class and on the playground. | Self-empowerment is magical. Children do better in their studies because THEY have the power to accomplish; they're not as dependent on adults for their success. | Parents have got to realize confidence in their children succeeding, whether it's knowing their kid won't be a bully/be bullied, that the child knows what to do if he/she gets separated in a store for instance, or what to do in a personal emergency. | Going forward, *radKIDS®* will be less likely to engage in personally dangerous behaviors. The scope of this is over-arching for a community: less crime, more successful citizens, etc. |
| Safety of the child | Less bullying | How to stop bullying and safety. | Safety of child | Safer community |
| A great help to the reduction of bullying behavior in the school. | Help kids with strategies for bullies. | Everything! | Having the parent book really helps parents know the transparency of the program, which encourages support. | Community changing principles! |
